# Adipose Derived-Mesenchymal Stem Cells Viability and Differentiating Features for Orthopaedic Reparative Applications: Banking of Adipose Tissue

**DOI:** 10.1155/2016/4968724

**Published:** 2016-11-29

**Authors:** Ilaria Roato, Daniela Alotto, Dimas Carolina Belisario, Stefania Casarin, Mara Fumagalli, Irene Cambieri, Raimondo Piana, Maurizio Stella, Riccardo Ferracini, Carlotta Castagnoli

**Affiliations:** ^1^CeRMS, A.O.U. Città della Salute e della Scienza, Torino, Italy; ^2^Skin Bank, Department of General and Specialized Surgery, A.O.U. Città della Salute e della Scienza, Torino, Italy; ^3^Department of Orthopaedic Oncology, CTO Hospital, Torino, Italy

## Abstract

Osteoarthritis is characterized by loss of articular cartilage also due to reduced chondrogenic activity of mesenchymal stem cells (MSCs) from patients. Adipose tissue is an attractive source of MSCs (ATD-MSCs), representing an effective tool for reparative medicine, particularly for treatment of osteoarthritis, due to their chondrogenic and osteogenic differentiation capability. The treatment of symptomatic knee arthritis with ATD-MSCs proved effective with a single infusion, but multiple infusions could be also more efficacious. Here we studied some crucial aspects of adipose tissue banking procedures, evaluating ATD-MSCs viability, and differentiation capability after cryopreservation, to guarantee the quality of the tissue for multiple infusions. We reported that the presence of local anesthetic during lipoaspiration negatively affects cell viability of cryopreserved adipose tissue and cell growth of ATD-MSCs in culture. We observed that DMSO guarantees a faster growth of ATD-MSCs in culture than trehalose. At last, ATD-MSCs derived from fresh and cryopreserved samples at −80°C and −196°C showed viability and differentiation ability comparable to fresh samples. These data indicate that cryopreservation of adipose tissue at −80°C and −196°C is equivalent and preserves the content of ATD-MSCs in Stromal Vascular Fraction (SVF), guaranteeing the differentiation ability of ATD-MSCs.

## 1. Introduction

In the last years many studies have been focused on tissue regeneration and reparative medicine, particularly to cure osteoarthritis, characterized by loss of articular cartilage also due to a reduced chondrogenic activity of mesenchymal stem cells (MSCs) from patients [1]. Adult MSCs, originally retrieved from bone marrow [2], can also be isolated from adipose tissue with higher frequency than in bone marrow [3] and show a great seductive potential to the orthopaedic community for their reparative capabilities and differentiation ability towards adipogenic, osteogenic, and chondrogenic lineages [4, 5], to home in injured tissues, to release factors for wound healing, and to modulate the immune system [5–7]. Even though adipose tissue is mainly constituted by adipocytes, it also contains the Stromal Vascular Fraction (SVF) that comprehends adipose tissue-derived MSCs (ATD-MSCs), endothelial cells, pericytes, fibroblasts, and hematopoietic-lineage cells [4, 5, 8, 9]. ATD-MSCs express many markers common to bone marrow-derived stem cells [10, 11], such as CD90, CD44, CD73, CD105, and CD271 [5, 12]. In particular, CD271 has been proposed as marker of primary choice for tissue regeneration by ATD-MSCs in older subjects, since this subpopulation is maintained in elderly people [13], and CD271+ MSCs are associated with a high efficiency of proliferation and trilineage differentiation compared to CD271− MSCs [12, 14].

The treatment of symptomatic knee arthritis with ATD-MSCs proved effective with a single infusion [15–19]; the efficacy of multiple infusions of ATD-MSCs in the articular joint is considered but still debated due to lack of data from clinical trials [19]. The availability of banking adipose tissue would allow treating with multiple infusions patients, avoiding repeated liposuction procedures for patients. Until now, many studies have been done to define protocols of banking able to guarantee effective quantity and quality of ATD-MSCs [20–24], without a conclusive statement. In the present work, we investigated banking procedures of adipose tissue, studying ATD-MSCs viability and differentiation capability after cryopreservation. In attempt to define the best condition for cryopreservation of adipose tissue, we analysed different parameters affecting it. In particular, confirming literature data [25], we reported that the presence of anesthetic during lipoaspiration negatively affects cell viability of cryopreserved adipose tissue and cell growth of ATD-MSCs in culture. Between the two cryoprotectant tested agents, we observed that DMSO guarantees a faster growth of MSCs in culture than trehalose. At last, ATD-MSCs derived from fresh and cryopreserved samples at −80°C and −196°C showed viability and differentiation ability comparable to fresh samples. These data indicate that cryopreservation of adipose tissue at −80°C and −196°C is equivalent and preserves the content of ATD-MSCs in SVF, guaranteeing the differentiation ability of ATD-MSCs.

## 2. Methods

### 2.1. Sample Collection

A total number of 80 lipoaspirate samples were collected after informed written consent of donors, according to the local ethic committee. Thirty-five patients were treated with Klein solution (lidocaine 1%, sodium bicarbonate 10 mEq, and adrenaline 1 mg/mL in 500 mL of solution of NaCl 0.9%) and 23 patients were treated with a solution without anesthetic (adrenaline 1 mg/mL in 500 mL of solution of NaCl 0.9%). Since the analysis between these two groups showed that the presence of anesthetic reduced MSC growth, the last 22 patients were collected without anesthetic.

Low-pressure liposuction with fenestrated blunt cannulae, according to Coleman procedure, was used to harvest adipose tissue [26, 27]. The lipoaspirates were centrifuged at 3000 rpm for 3 minutes to collect the fat phase and then washed twice with PBS by centrifugation at 1700 rpm for 10 minutes. The fat phase was processed either for the analysis of cell components or for cryopreservation.

### 2.2. Cryopreservation and Viability Assay of Adipose Tissue

Fat phase was frozen in 5 mL cryotubes with 2 different cryoprotective solutions: (i) fetal bovine serum (FBS: Fetal Bovine Serum, certificated-EDQ, Bio West SAS) + 10% dimethyl sulfoxide (DMSO-Cry-on, AL.CHI.MI.A.Srl), *n* 30 and (ii) FBS + 0,35 M trehalose (Sigma-Aldrich), *n* 34. Trehalose is a valid alternative to DMSO because it is not toxic for cells at normal body temperature. Samples were mixed with a cool cryopreservative solution and then transferred in freezing container, which guarantees a 1°C/min cooling rate, required for successful cryopreservation of cells and put at −80°C for 3 days, and then part of the sample was stored in liquid nitrogen (−196°C).

The frozen tissues were fast thawed in a water bath at 37°C; the cryopreservation solution was diluted with medium supplemented with 10% FBS and centrifuged to remove the CPA.

### 2.3. Isolation of SVF from Adipose Tissue

SVFs were isolated from fresh and thawed adipose tissue by enzymatic digestion with Collagenase NB4 (Serva Electrophoresis) 0,3 U/mL using a tube rotator for 40 minutes at 37°C. The activity of enzyme was neutralized by the addition of DMEM low glucose + 10% FBS, and then the samples were centrifuged at 1700 rpm for 10 minutes. The pellet was resuspended in medium, then passed through 100 *μ*m and 70 *μ*m cell strainers, washed by saline solution, collected, and counted. The phenotype of MSCs contained in the SVF was evaluated at T0, soon after SVF isolation. The SVF cells were seeded in T25 flasks and cultured in DMEM with 10% FBS, 2 mM glutamine, and 1% antibiotics (Gibco, Life Technologies) and the medium was replaced to eliminate nonadherent cells after 24 h. After 7 days of culture, the growth rate was calculated by counting the number of cells grown for mL of adipose tissue. Then MSCs were cultured for 2 passages; then their phenotype was analysed by flow cytometry.

### 2.4. Phenotype of ATD-MSCs

Cell surface markers of ATD-MSCs were analysed by flow cytometry on fresh SVF and on SVF derived after thawing of adipose tissue and its collagenase treatment. ATD-MSCs were identified as CD105, CD44, CD73, and CD271 positive cells and negative for CD45 expression. Standard labelling protocol was performed with the following antibodies fluorochrome-conjugated and isotypic controls: human CD105 PE (Invitrogen), CD73 FITC (kindly provided by Professor Malavasi, University of Turin), CD44 FITC, CD45 PerCP, CD3 PerCP, CD271 APC, IgG1 PE, IgG1 APC, and IgG2a PerCP (Miltenyi Biotec), and IgG1 FITC-conjugated (Immunostep). About 10^5^ events/sample were used for capture with CellQuest software. All data were analysed with FlowJo software (Tree Star).

### 2.5. Tissue Biopsies and Staining Procedures

All fresh and preserved samples were processed for staining procedures. Adipose tissue samples were snap frozen in isopentane quenched in liquid nitrogen, mounted in OCT 4583 embedding compound (Sakura), and stored at −80°C. Ten-micron-thick cryostat sections were cut and transferred to polylysine-coated slides. The slides were air dried for two hours and then processed. For histological analysis a section from each biopsy was counterstained with Mayer's hematoxylin solution (DAKO) and mounted with an aqueous mounting medium (Kaiser's glycerol gelatin, from MERCK), to confirm the adequacy of the specimen. Slides were examined double blindly; microphotographs were taken using Leica DMLA microscope.

For immunohistochemical analysis (IHC) the sections were incubated with primary mouse monoclonal antibodies (MoAb) CD31 (Clone 10G9 ABCAM; 1 : 100), CD105 (Clone 266 BD PHARMINGEN; 1 : 50), and CD68 (Clone EBM11 DAKO; 1 : 500). They were titrated to yield maximal specific staining and minimal nonspecific or background staining. The endogenous peroxidase activity was inhibited by the addition of methyl alcohol and 0.03% hydrogen peroxide. A second incubation with biotinylated secondary antibody and an avidin-biotin-horseradish peroxidase complex LSAB + (DAKO) were performed. Staining was developed with 3-amino-9-ethyl carbazole as a chromogen. All samples were counterstained with Mayer's hematoxylin solution (DAKO) and mounted with Kaiser's glycerol gelatin. Slides were examined double blindly and microphotographs were taken using a DMLA Leica microscope equipped with a digital camera (Leica DFC 425C). Images were acquired using LAS software (Leica Application Suite).

Immunofluorescence staining with (Moab) Aggrecan (Clone 969D4D11 Invitrogen; 1 : 25) was performed using appropriate Rhodamin (Chemicon 1 : 100) conjugated isotype-specific secondary antibodies. Slides were examined double blindly; microphotographs were taken using a DMLA Leica microscope equipped with a digital camera (Leica DFC 425C). Images were acquired using LAS software (Leica Application Suite).

### 2.6. MTT on Adipose Tissue Samples

Ten samples with similar weight, including 2 negative controls, were chosen and tested from each adipose tissue specimen. MTT salts solution (0.5 mg/mL) was added and the samples were then incubated at 37°C and 5% CO_2_. After 3 hrs of incubation the precipitate salts were solubilized for 3 hrs with the use of 2-methoxyethanol (Sigma-Aldrich). The solution was then read on a spectrophotometer (570 nm). A negative control was always set up in double (a heat-denatured specimen for 20 minutes at maximum temperature) for each experimental condition. The negative control was then treated as other samples and optical density (OD) subtracted from each sample.

Adipose tissue viability of the adipose sample was expressed as viability index, deriving by the ratio between the samples' OD and their weight in grams. Noncryopreserved fresh tissue samples were used as control group. The formulas used in the determinations were Index of Viability (I.V.) = optical density (595 nm)/grams of tissue. Percent viability (% viability) = (I.V. of cryopreserved samples/I.V. of control fresh samples) × 100.

### 2.7. Evaluation of Chondrogenic and Osteogenic Potential of ATD-MSCs Derived from Fresh and Thawed Samples

We cultured SVF in DMEM low glucose supplemented with 10% FBS to obtain a population of ATD-MSCs, which were then cultured with StemPro Chondrogenesis Differentiation kit (Gibco, Life technologies) according to the manufacturers' instructions to induce chondrogenesis. After 21 days, cells were stained for immunofluorescence by Aggrecan. To study osteogenesis we plated ATD-MSCs in differentiating medium according to Brunetti et al. [28] for 14 days, and then we stained them for alkaline phosphatase according to kit produced by Sigma-Aldrich. The mineralization activity of osteoblasts was studied by culturing ATD-MSCs in alpha-MEM supplemented with 10% FBS, 50 *μ*g/mL ascorbic acid, 10^−8 ^M dexamethasone, and 10 mM beta-glycerophosphate (Sigma-Aldrich) for 8 weeks. The formation of mineralized nodules was accessed by von Kossa staining.

### 2.8. Statistical Analysis

All statistical analysis was carried out using GraphPad Prism 7. Data were presented as mean with standard error and calculated by One-way Anova with multiple comparisons by Bonferroni test. Results were considered significant with *p* < 0.05. For evaluation of the growth rate we used Mann-Whitney test.

## 3. Results

### 3.1. Local Anesthetic Reduces ATD-MSC Growth

**Figure 1:**
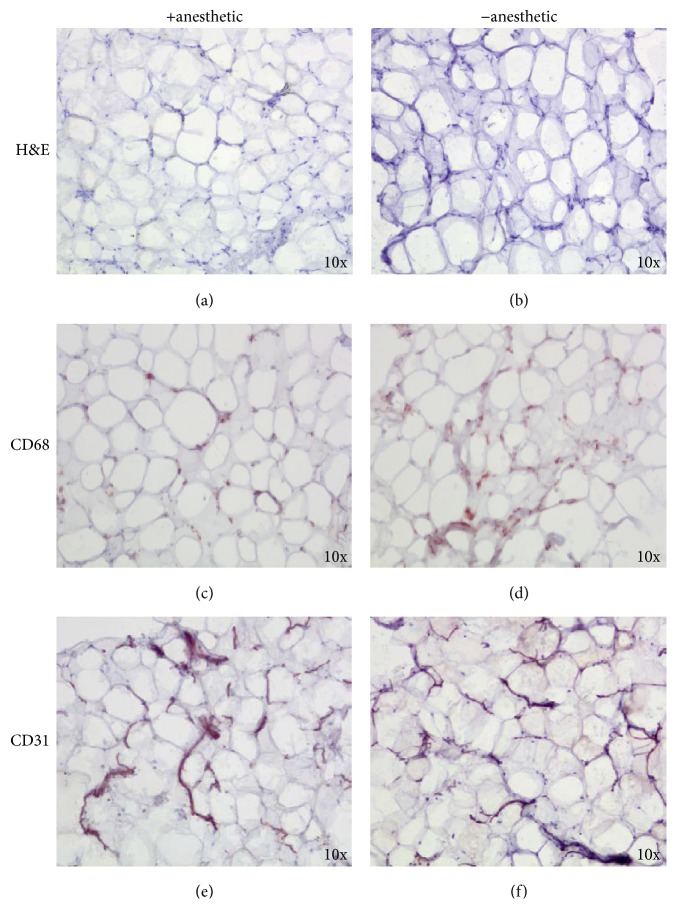
Local anesthetic did not interfere with adipose tissue morphology. H&E staining showed a similar morphology of adipose tissue harvested with or without local anesthetic. No evidence of adipose tissue degeneration or necrosis was evident (a, b). IHC analysis showed comparable staining for CD68 and CD31, in both samples harvested with or without anesthetic (c–f).

**Figure 2:**
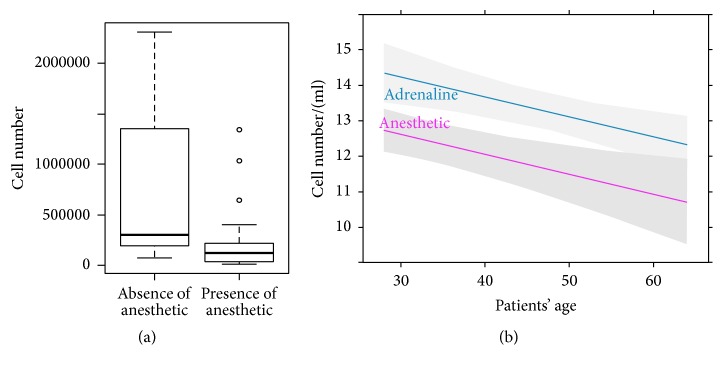
Local anesthetic reduced ATD-MSCs growth. (a) Box and whisker plot showed a significant difference in the number of cells isolated from adipose tissues harvested with or without anesthetic. (b) The regression analysis showed that, after 7 days, the number of cells grown in culture was reduced when the anesthetic was added to the solution of infiltration, particularly in relation to the increasing of the patients' age, *p* < 0.001.

Adipose tissues harvested with (*n* 35)/without (*n* 23) local anesthetic maintained normal histological structure without evident differences between 2 groups. Both with and without anesthetic, the tissues were compact, with polygonal cells and good stroma, without any evidence of fatty tissue degeneration or necrosis (Figures 1(a) and 1(b)). These data were also confirmed by the IHC analysis, showing that the different cellular components of adipose tissue, stained with specific antibodies, resulted comparably in all analysed samples (Figures 1(c)–1(f)). Despite the absence of effects on cell morphology, the presence of the anesthetic significantly reduced the number of cells isolated from adipose tissues harvested with or without anesthetic (Figure 2(a)). Moreover, after 7 days of* in vitro* culture, we observed that cell growth was reduced when anesthetic was present in the infiltration solution, particularly in relation to the increase of the patients' age, *p* < 0.001 (Figure 2(b)).

### 3.2. DMSO Is Better Than Trehalose for Cryopreservation of Adipose Tissue

**Figure 3:**
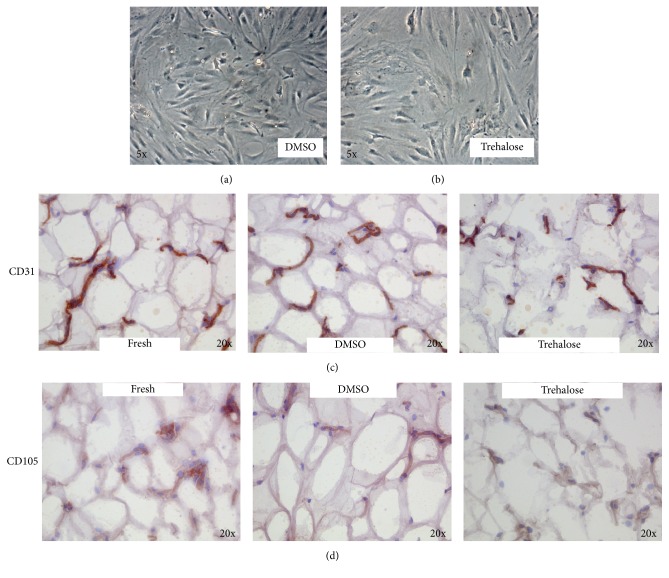
DMSO cryopreserved adipose tissue better than trehalose. ATD-MSCs isolated from DMSO cryopreserved lipoaspirates showed a better morphology compared with trehalose ((a) and (b)). The IHC staining for CD31 (line (c)) and CD105 (line (d)) showed that the different cellular structures were better preserved in samples cryopreserved with DMSO than with trehalose.

As cryoprotectant agents (CPA) for adipose tissue, we tested DMSO and trehalose, showing a comparable viability of samples with both CPA after thawing (Table 1). In contrast, cell cultures showed that ATD-MSCs isolated from lipoaspirates cryopreserved in DMSO had a more rapid growth and arrived at confluence in a few days with a better morphology as opposed to the cells with trehalose (Figures 3(a) and 3(b)). The morphological analysis and the staining for CD31 and CD105 showed that the cellular structures were better preserved in samples cryopreserved with DMSO than with trehalose (Figures 3(c) and 3(d)).

### 3.3. Cryopreservation of Lipoaspirates at Both −80°C and −196°C Guarantees Viability of Adipose Tissue and ATD-MSCs

**Figure 4:**
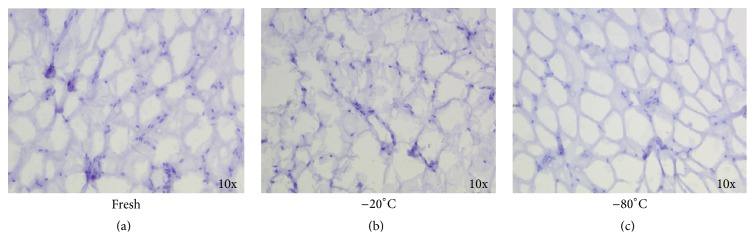
Adipose tissue is better preserved at −80°C than −20°C. The samples cryopreserved at −20°C (b) showed a damaged structure compared to fresh (a) and −80°C (c).

**Figure 5:**
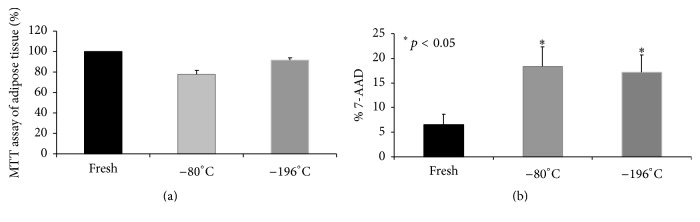
Cryopreservation at −80°C and −196°C guaranteed viability of adipose tissue and ATD-MSCs. The percentages of viability of adipose tissue normalized on fresh sample are reported (a). 7-AAD staining showed a significant increase of dead cells in cryopreserved compared to fresh samples, *p* < 0.05, but there was not any difference between the two temperatures of cryopreservation (b).

**Figure 6:**
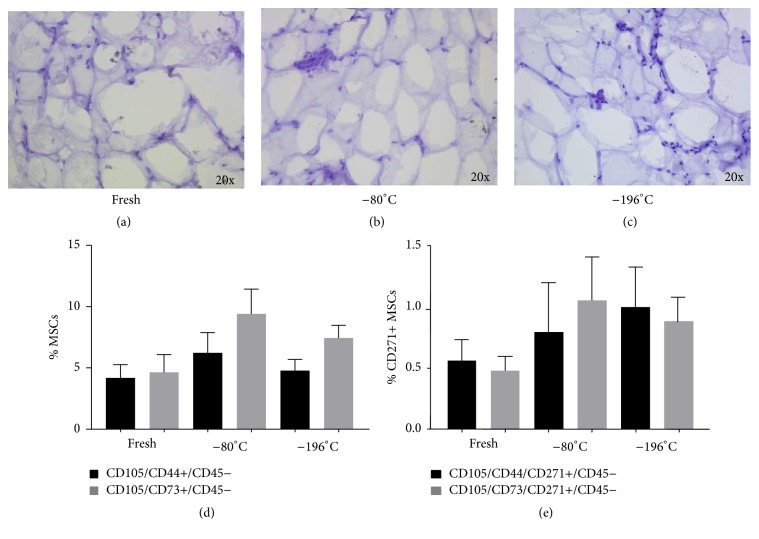
Cryopreservation at −80°C and −196°C maintained morphology of adipose tissue and the content of ATD-MSCs. The morphology of cryopreserved adipose tissue at −80°C and −196°C was maintained, without evidence of tissue degeneration or necrosis, comparable to the fresh sample (a–c). The percentage of CD105, CD44, and CD73+ ATD-MSCs and the CD271+ subpopulation was comparable to fresh samples and also between the two temperatures (d, e).

In a first series of cases, to define the best temperature of cryopreservation for adipose tissue, we evaluated by MTT assay the viability of tissue derived from lipoaspirates after cryopreservation at −20°C and −80°C. Fat preserved at lower temperature showed a greater viability index (I.V.) than samples preserved at −20°C, Table 2. The samples preserved at −20°C showed a damaged structure and the tissue resulted in suffering compared to fresh and −80°C (Figure 4). Due to the low quality of preservation at–20°C, we then decided to compare −80°C and −196°C. The percentages of viability for adipose tissue after thawing were 77,6% and 91,6% for samples cryopreserved at −80°C and −196°C, respectively (Figure 5(a)). The mean I.V. values were reported in Table S1 (in Supplementary Material available online at http://dx.doi.org/10.1155/2016/4968724). The analysis of cell mortality in the SVF, derived after collagenase digestion of adipose tissue, showed a major number of dead cells in cryopreserved (17% of 7-AAD+ cells) compared to fresh samples (7% of 7-AAD+ cells), *p* < 0.05, but again there was not any difference between the two temperatures of cryopreservation (Figure 5(b)). To further confirm this result, the morphology of cryopreserved adipose tissue at −80°C and −196°C showed well-maintained structures, without evidence of tissue degeneration or necrosis, comparable to the fresh sample (Figures 6(a)–6(c)).

We analysed a panel of standard surface markers for MSCs to study the phenotype of SVF derived from fresh and thawed adipose tissue, showing that the percentage of CD105/CD44/CD73+/CD45− cells and the subpopulation also expressing CD271 were comparable between the two temperatures and with fresh sample (Figures 6(d) and 6(e)). Thus the cryopreservation at both −80°C and −196°C did not interfere with the content of ATD-MSCs.

### 3.4. Cryopreservation of Lipoaspirates at Both −80°C and −196°C Preserves Differentiating Abilities of ATD-MSCs* In Vitro*

**Figure 7:**
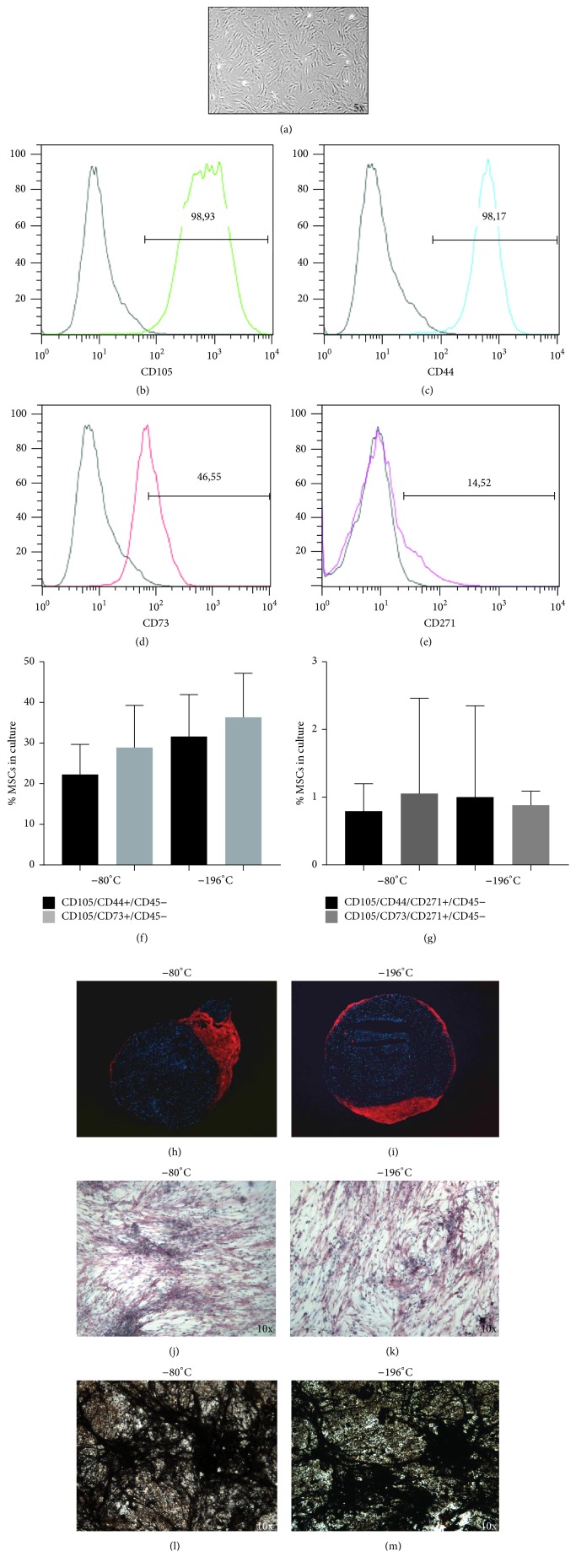
ATD-MSCs differentiating abilities* in vitro* were preserved at both temperatures of cryopreservation. After cell culture, ATD-MSCs assumed the typical fibroblast-like morphology (a) and expressed CD105, CD44, CD73, and CD271 (b–e). The immunophenotype of MSCs expressing CD105/CD44/CD73 and of the subpopulation CD105/CD44/CD73/CD271+ cells was comparable with both the temperatures of cryopreservation (f, g). Chondrocytes (h, i) and osteoblasts (j, k) formed from both adipose tissues cryopreserved at −80°C and –196°C. von Kossa staining showed that osteoblasts had comparable ability to mineralize at both temperatures (l, m).

We cultured* in vitro* SVF cells derived from collagenase treatment of adipose tissue both fresh and thawed, showing the proliferation of cell with the typical fibroblast-like morphology (Figure 7(a)), that expressed mesenchymal markers such as CD105, CD44, CD73, and CD271 (Figures 7(b)–7(e)), whereas they were negative for hematopoietic markers (data not shown). In these cultures, we observed a selective growth of mesenchymal population expressing CD105/CD44/CD73 and of CD105/CD44/CD73/CD271+ subpopulation, comparable between the two temperatures of cryopreservation (Figures 7(f) and 7(g)). To test whether cryopreservation affects the chondrogenesis and osteogenesis capabilities of these ATD-MSCs, we cultured them in specific media, showing formation of chondrocytes and osteoblasts, both from adipose tissue cryopreserved at −80°C and −196°C (Figures 7(h)–7(k)). We also tested the activity of these osteoblasts, showing their ability to mineralize (Figures 7(l) and 7(m)).

## 4. Discussion

Regenerative and in particular reparative medicine represent the new frontier for orthopaedic diseases, such as osteoarthritis, that can benefit of treatments with ATD-MSCs. Indeed, to date, clinical studies suggest that SVF and ATD-MSCs are safe and capable of tissue repairing [15, 18, 19, 29, 30]. Various well-defined harvesting techniques are available for liposuction procedure [27, 31, 32], even though there is not one method providing the best results in terms of viability and volume of adipose tissue with its cellular components. Previously published data reported a marked influence of local anesthetics on the quantity and quality of viable preadipocytes [25, 33]. Our results showed that lidocaine seems not to affect morphology of adipose tissue, but the derived ATD-MSCs reduced their growth in cultures* in vitro*. These data were in accordance with the literature, which also showed a reduced ATD-MSC viability [34] and chondrocyte cytotoxicity due to local anesthetics [35], prompting us to suggest performing liposuction without local anesthesia.

Literature data reported different methods of cryopreservation for human adipose tissue, investigating both the use of different CPAs and temperatures [21, 36, 37]. Different CPAs alone or in combinations can improve the viability of this tissue during the procedure of freezing and thawing [38], since CPAs reduced the osmotic stress. Here we tested DMSO and trehalose, showing a viability of samples comparable to fresh ones, with both CPAs after thawing. Nonetheless, ATD-MSCs isolated from lipoaspirates cryopreserved in DMSO grew faster and with a better morphology than the cells derived from trehalose cryopreservation. Even though DMSO is known for its cytotoxicity and its clinical use is limited, it gave us the best results, and thus we are considering utilizing the GMP grade DMSO, which is available for clinical use.

The optimal temperature for cryopreservation of adipose tissue is still under investigation; indeed some works report that −20°C is comparable to liquid nitrogen for a short term storage of adipose tissue [36, 37]. The length of the cryopreservation period is particularly relevant, since to allow multiple infusions it is necessary to store adipose tissue for long time, and thus it is mandatory to investigate both different CPAs and temperatures for longer times. We started to thaw one month after freezing, showing a significant reduced viability of lipoaspirates at −20°C compared to −80°C, according to Wolter et al. [39]. Since Moscatello et al. examined storage at −20°C and liquid nitrogen, suggesting that the last is better [40], we decided to compare −80°C and −196°C to evaluate viability of adipose tissue and above all the maintenance of ATD-MSC ability to differentiate into osteoblasts and chondrocytes. Literature reports only one study that contemporarily compared −20°C, −80°C, and liquid nitrogen temperatures, which did not show any differences in cell viability about the three temperatures. Nonetheless, in this work the period of storage was short and their aim was to examine the viability of adipose tissue and adipocytes for fat graft in plastic surgery [36]. Here, we report the results on adipose tissue viability and differentiation capabilities of ATD-MSCs towards osteoblasts and chondrocytes, after one month of cryopreservation at −20°C, −80°C, and −196°C. We demonstrated that the I.V. of samples cryopreserved at −80°C and in liquid nitrogen was comparable to fresh samples, whereas at −20°C the viability was reduced. We also observed that no differences were present between samples cryopreserved at −80°C and −196°C, thawing after two months (data not shown).

Among the different markers available for the characterization of MSCs, described by the Mesenchymal and Tissue Stem Cell Committee of the International Society for Cellular Therapy [41], we tested positivity for CD105, CD44, CD73, and the contemporary negativity for CD45. We also studied the expression of CD271, since CD271+ ATD-MSCs are associated with a high efficiency of proliferation and trilineage differentiation compared to CD271− counterpart [12]. Moreover, even though a decrease in stem cell number is typical in elderly people, the subpopulation of MSCs expressing CD271 is also reduced during ageing [13, 42]; however, they are always present in all age groups according to Cuevas-Diaz Duran et al. [13], and thus CD271 has been proposed as marker of primary choice for tissue regeneration by ATD-MSCs in older subjects. We reported that cryopreservation did not alter the frequency of CD105/CD44/CD73+/CD45− cells and of the subpopulation expressing CD271, in SVFs derived from fresh and thawed samples. This result is relevant since even though the percentage of dead cells was slightly increased in thawed samples compared to the fresh ones, the cryopreservation both at −80°C and at −196°C did not interfere with the content of ATD-MSCs, likely due to a protective action of adipose tissue on these cells.

ATD-MSCs derived from cryopreserved samples at both −80°C and −196°C differentiated into chondrocytes and osteoblasts, which showed also mineralization activity. Thus, according to literature [20], ATD-MSCs derived from cryopreserved adipose tissue were functionally equivalent to freshly isolated ones.

In conclusion, the creation of an Autologous Adipose Tissue Bank would avoid repeated lipoaspiration procedures for patients to guarantee the availability of SVF for multiple injections. The demonstration that cryopreservation of adipose tissue, for long time, allows maintaining the viability of SVF, particularly of ATD-MSCs, would allow testing the efficacy of multiple infusions of SVF in treatment of patients with osteoarthritis. This could be an alternative approach to the expansion* ex vivo* of ATD-MSCs, since a direct transfer of the cellular component from the donor to the acceptor site of the same patient is a simple and straightforward method, avoiding the complexity of constructs and* ex vivo* cell cultures performed in laboratory.

## Supplementary Material

Table S1 Viability Index of Adipose Tissue. The mean IV value ± SD was reported at T0 (fresh sample) and at both cryopreservation temperatures. The IV values were similar between the two temperatures.

## Figures and Tables

**Table 1:** Viability index with different cryoprotectants. Viability indexes (I.V.) was similar between the two CPAs.

	DMSO	Trehalose
	*N* = 30	*N* = 34
Age	47 (37–54)	47 (42–57)
I.V. of fresh samples	12,3 (10,1–14,8)	14,7 (11,2–16,7)
I.V. after cryopreservation	6,48 (5,45–9,40)	6,59 (5,73–7,79)

**Table 2:** Viability index at different temperatures of cryopreservation. The viability index (I.V.) of cryopreserved samples was significantly higher at −80°C compared to −20°C, *p* < 0.05.

	−20°C	−80°C
	*N* = 25	*N* = 39
Age	46 (36–53)	49 (42–57)
I.V. of fresh samples	12 (9,6–13,1)	15,8 (11,5–16,8)
I.V. after cryopreservation	5,6 (5,2–7,1)	7,2^*∗*^ (5,6–10,3)

The viability index (I.V.) of cryopreserved samples was significantly higher at −80°C compared to −20°C; *∗* refers to *p* value.
