# Supplementary material for: Adipose Derived-Mesenchymal Stem Cells Viability and Differentiating Features for Orthopaedic Reparative Applications: Banking of Adipose Tissue
Source: Stem Cells Int. 2016 Nov 29;2016:4968724. doi: 10.1155/2016/4968724 (PMC5153503; doi:10.1155/2016/4968724)
Supplement: Supplementary file 1 — Table S1 Viability Index of Adipose Tissue. The mean IV value ± SD was reported at T0 (fresh sample) and at both cryopreservation temperatures. The IV values were similar between the two temperatures. [file 4968724.f1.doc]

### **Table S1 Viability Index of Adipose Tissue**

I.V. 15,5± 2,3

(15,1-18,2)

I.V. 13,2± 4

(7,1-17)

I.V. 17,05± 6

(7,3-26,9)

**-196°C**

N=10

**-80°C**

N=10

**fresh (T0)**

The mean IV value ± SD at T0 and at both cryopreservation temperatures are reported
